# Non-stenotic Carotid Plaques in Embolic Stroke of Unknown Source

**DOI:** 10.3389/fneur.2021.719329

**Published:** 2021-09-22

**Authors:** Joseph Kamtchum-Tatuene, Ali Z. Nomani, Sarina Falcione, Danielle Munsterman, Gina Sykes, Twinkle Joy, Elena Spronk, Maria Isabel Vargas, Glen C. Jickling

**Affiliations:** ^1^Faculty of Medicine and Dentistry, Neuroscience and Mental Health Institute, University of Alberta, Edmonton, AB, Canada; ^2^Department of Medicine, Faculty of Medicine and Dentistry, University of Alberta, Edmonton, AB, Canada; ^3^Division of Neuroradiology, Department of Radiology and Medical Imaging, Geneva University Hospital, Geneva, Switzerland

**Keywords:** stroke, carotid stenosis, carotid plaque, biomarkers, atherosclerosis

## Abstract

Embolic stroke of unknown source (ESUS) represents one in five ischemic strokes. Ipsilateral non-stenotic carotid plaques are identified in 40% of all ESUS. In this narrative review, we summarize the evidence supporting the potential causal relationship between ESUS and non-stenotic carotid plaques; discuss the remaining challenges in establishing the causal link between non-stenotic plaques and ESUS and describe biomarkers of potential interest for future research. In support of the causal relationship between ESUS and non-stenotic carotid plaques, studies have shown that plaques with high-risk features are five times more prevalent in the ipsilateral vs. the contralateral carotid and there is a lower incidence of atrial fibrillation during follow-up in patients with ipsilateral non-stenotic carotid plaques. However, non-stenotic carotid plaques with or without high-risk features often coexist with other potential etiologies of stroke, notably atrial fibrillation (8.5%), intracranial atherosclerosis (8.4%), patent foramen ovale (5–9%), and atrial cardiopathy (2.4%). Such puzzling clinical associations make it challenging to confirm the causal link between non-stenotic plaques and ESUS. There are several ongoing studies exploring whether select protein and RNA biomarkers of plaque progression or vulnerability could facilitate the reclassification of some ESUS as large vessel strokes or help to optimize secondary prevention strategies.

## Introduction

Ischemic stroke is considered cryptogenic when no definite cause is identified during the baseline etiological workup (1). According to the Cryptogenic Stroke/Embolic Stroke of Undetermined Source International Working Group, the baseline etiological workup should include brain imaging with computed tomography (CT) or magnetic resonance imaging (MRI), assessment of the heart rhythm with 12-lead ECG and continuous cardiac monitoring for at least 24 h with automated rhythm detection, transthoracic cardiac ultrasound, and imaging of cervical and intracranial vessels supplying the infarcted brain region (using CT, MRI, conventional angiography, or ultrasonography) (2).

**Figure 1:**
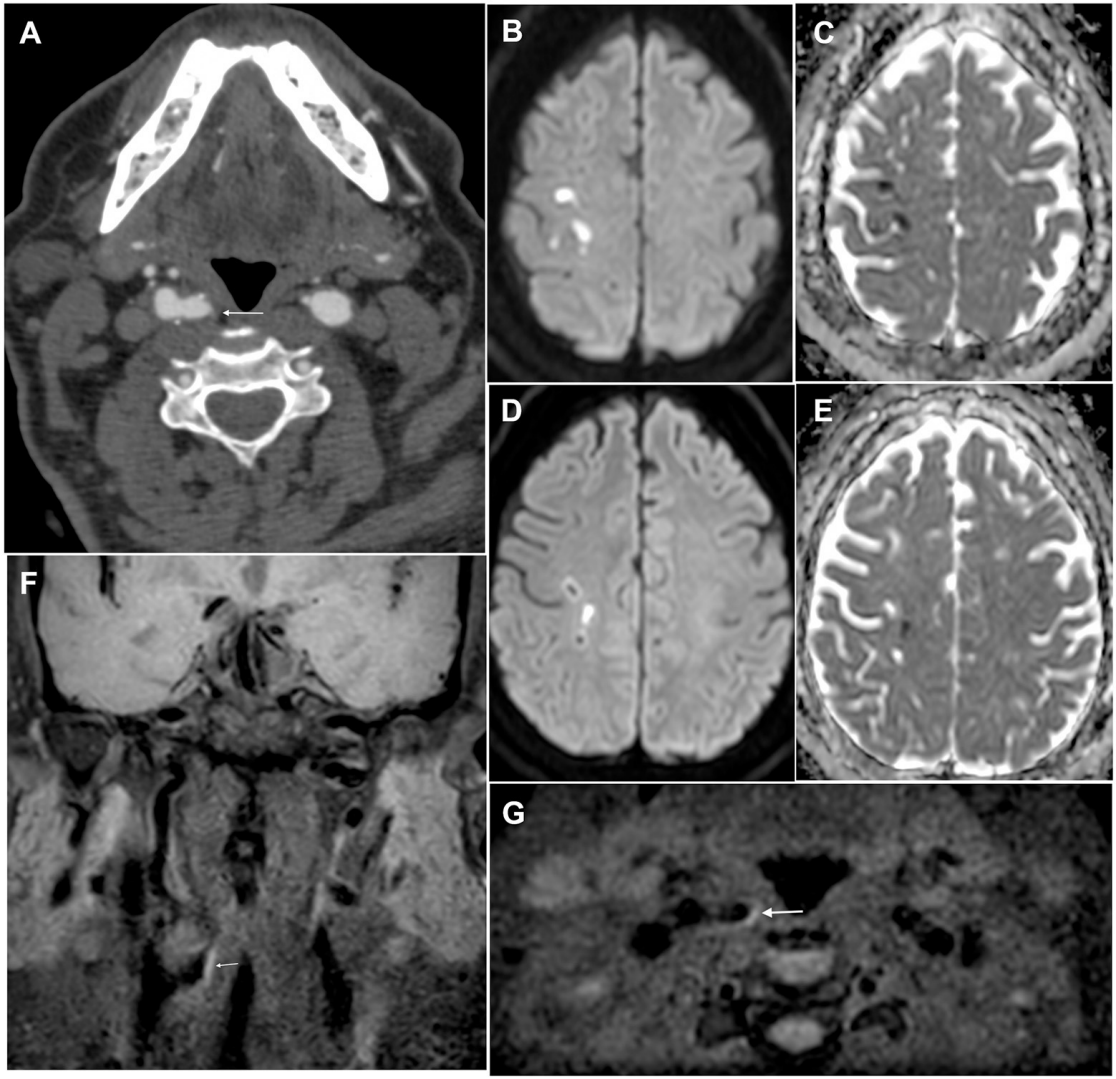
Brain and plaque imaging findings in a 64-year-old man with ESUS. **(A)** Axial angio-CT scan slice showing a hypodense non-stenotic carotid plaque in the right internal carotid artery (white arrow). **(B–E)** Axial diffusion-weighted imaging slices (with corresponding ADC maps) showing multiple embolic strokes in the right pre-and post-central area. **(F,G)** Coronal and axial T1-weighted black blood sequence showing hyperintensity of the non-stenotic plaque in the right internal carotid artery (white arrow), thus confirming the presence of intraplaque hemorrhage.

Cryptogenic strokes represent ~30% of all ischemic strokes. They could be further classified into three subgroups: stroke with no cause despite complete baseline workup, stroke with multiple possible underlying causes, and stroke with incomplete baseline workup (3). In the subgroup of cryptogenic strokes with complete workup, embolic stroke of unknown source (ESUS) is a clinical construct referring to non-lacunar ischemic strokes (size >1.5 cm on CT or >2.0 cm on diffusion MRI) of presumable embolic origin (superficial/cortical brain lesion) despite the absence of any obvious sources of cardiac or arterial embolism (e.g., atrial fibrillation, carotid, or intracranial stenosis > 50%) (Figure 1) (2). ESUS represent ~17% of all ischemic strokes with a recurrent stroke rate of 4.5% per year despite antithrombotic therapy (4–6).

The definition of ESUS was based on the assumptions that cryptogenic strokes may be related to covert atrial fibrillation and that a relationship between non-stenotic atherosclerotic plaques (causing <50% stenosis) and stroke was unlikely. However, there is now evidence to suggest that ESUS represents a heterogeneous group including patients with various other potential causes of stroke besides atrial fibrillation (7–9). Such causes include atrial cardiopathy (10), patent foramen ovale (PFO) (11), cancer (12), and non-stenotic plaques affecting the aortic arch or carotid, vertebral, or intracranial arteries (7, 13, 14). Atrial cardiopathy is a concept referring to a dysfunction of the left atrium that is thought to favor and precede the onset of atrial fibrillation and its eventual detection by electrocardiographic devices. The diagnosis is based on the identification of imaging markers (e.g., left atrial enlargement, spontaneous echocontrast in the left atrium or the left atrial appendage, atrial fibrosis with delayed gadolinium enhancement on MRI), electrocardiographic markers (e.g., paroxysmal supraventricular tachycardia, increased P-wave terminal force in V1, interatrial block, prolonged PR), and blood biomarkers (e.g., N-terminal pro-brain natriuretic peptide, highly sensitive cardiac troponin T) (10).

Non-stenotic carotid plaques are found in 40% of patients with ESUS and 10–15% of patients with ESUS have mild stenosis (20–49%) (2, 15–17). Here we review the evidence supporting the relationship between non-stenotic carotid plaques with high-risk features and stroke in patients with ESUS. We present the remaining challenges in the process of formally establishing the causal link between non-stenotic plaques and ESUS, notably those related to the identification of blood biomarkers of vulnerable plaque. Finally, we discuss the management of non-stenotic carotid plaques in patients with ESUS and highlight areas for future research.

## Non-stenotic Carotid Plaques as a Potential Cause of ESUS

The relationship between non-stenotic carotid plaques and ESUS is supported by a set of three clinical observations.

First, in patients with ESUS, carotid plaques are more prevalent on the side of the stroke than on the contralateral side. In a cross-sectional study of 85 patients with ESUS, non-stenotic carotid plaques thicker than 3 mm were present in 35% of ipsilateral carotid arteries vs. 15% of the contralateral carotid arteries (18). A similar finding was observed in a review of 138 ESUS cases from the prospective multicenter INTERRSeCT study (The Predicting Early Recanalization and Reperfusion With IV Alteplase and Other Treatments Using Serial CT Angiography). The investigators found a non-stenotic carotid plaque ipsilateral to the stroke in 29.2% of patients and contralateral to the stroke in 18.7% (17).

Second, in patients with ESUS, there is a lower incidence of atrial fibrillation detected during follow-up in patients with ipsilateral non-stenotic carotid plaques than in those without, thus suggesting that non-stenotic carotid plaques may be related to the stroke. In 777 participants of the New Approach Rivaroxaban Inhibition of Factor Xa in a Global Trial vs. ASA to Prevent Embolism in Embolic Stroke of Undetermined Source (NAVIGATE-ESUS) trial who were followed up for a median of 2 years, the incidence of atrial fibrillation was 2.9 per 100 person-years in patients with ipsilateral non-stenotic carotid plaques vs. 5.0 per 100 person-years in those without (overall rate: 8.5 vs. 19.0%; adjusted hazard ratio: 0.57, 95% CI 0.37–0.84) (15).

Third, plaques with high-risk features are more prevalent on the side of the stroke in patients with ESUS. In a meta-analysis of 8 studies enrolling 323 patients with ESUS, plaques with high-risk features were present in 32.5% of the ipsilateral carotid arteries vs. 4.6% of the contralateral carotid arteries. More specifically, the odds of finding a non-stenotic carotid plaque with a ruptured fibrous cap in the ipsilateral vs. the contralateral carotid artery was 17.5, reinforcing the idea that non-stenotic carotid plaques should not be considered as benign coincidental findings in patients with ESUS (13).

High-risk plaques have features on brain or vascular imaging that are associated with a higher risk of stroke in patients with either symptomatic or asymptomatic carotid atherosclerosis, independent of the grade of stenosis (19–24). The most common high-risk plaque features are echolucency, impaired cerebrovascular reserve, intraplaque hemorrhage (Figure 1), silent brain infarcts, lipid-rich necrotic core, large juxtaluminal black hypoechoic area, large plaque volume, plaque thickness, microembolic signals, mural thrombus, neovascularization, plaque irregularity, plaque inflammation or hypermetabolism, thin or ruptured fibrous cap, and ulceration (19, 21, 25–31). The American Heart Association combines some of these features to derive a classification of atherosclerotic plaques into 6 types reflecting increasing instability and risk of cardiovascular events (Table 1) (32–37). On average, high-risk plaque features are three times more prevalent in patients with symptomatic vs. asymptomatic carotid stenosis (OR = 3.4, 95% CI: 2.5–4.6) (19). They are detected using various vascular imaging modalities (Table 2). To date, there are no data on the risk of recurrent stroke associated with each of the high-risk features in patients with ESUS. Analysis of secondary outcome data from the Carotid Plaque Imaging in Acute Stroke study (CAPIAS; NCT01284933) might help to address this knowledge gap (35, 39).

**Table 1:** American Heart Association comprehensive morphological classification scheme for atherosclerotic lesions (32–34).

**Plaque type**	**Description**							
		**Lipid rich necrotic core**	**Fibrous cap**	**Calcification**	**Erosion/rupture**	**Intraplaque hemorrhage**	**Thrombus**	**Regression to normal**
Type I (Initial lesion)	Initial lesion, accumulation of smooth muscle cells and isolated foam cells, absence of a necrotic core.	Absent	Absent	Absent	Absent	Absent	Absent	Possible
Type II (Intimal xanthoma)	Multiple layers of foam cells, previously referred to as “fatty streak”	Absent	Absent	Absent	Absent	Absent	Absent	Possible
Type III (pre-atheroma)	Smooth muscle cells in a proteoglycan-rich extracellular matrix, multiple layers of foam cells, non-confluent extracellular lipid pools	Absent	Present (ill-defined)	Absent	Absent	Absent	Absent	Possible
Type IV (atheroma)	Confluent extracellular lipids	Present (well-formed)	Present (well-defined)	Absent	Absent	Absent	Absent	Not possible
Type Va (Fibroatheroma)	Confluent extracellular lipids with prominent proliferative fibromuscular layer	Present (well-formed)	Present (thick)	Possible^a^	Absent	Absent	Absent	Not possible
Type VI (Complicated atheroma)^*b*^	Inflammatory lesion with at least one high-risk feature	Present (large)	Present (thin or eroded)	Possible (partial calcification)	Possible (VIa if present alone)	Possible (VIb if present alone)	Possible (VIc if present alone)	Not possible

a *The plaque is assigned category Vb if predominantly calcified (fibro-calcific) or category Vc if predominantly fibrous (collagen-rich atheroma with smaller lipid core)*.

b *The plaque is assigned category VIabc if erosion/ulceration, intraplaque hemorrhage and luminal thrombus are present concurrently*.

**Table 2:** High-risk plaque features commonly used in clinical practice (13, 21, 25–31).

**High-risk plaque features^a^**	**Imaging modality of choice**	**Description^b^**	**Alternative imaging modalities**	**Prevalence (%)in patients with ESUS**
AHA type IV, V, VI (35–37)	MRI	Plaque with large lipid-rich necrotic core (>40% of the vessel circumference), ruptured fibrous cap, mural thrombus, or intraplaque hemorrhage (see below).	CT, US	In three studies including 82 patients with ESUS, an AHA plaque type IV-VI was found in the ipsilateral carotid in 38% of cases on average (35–37).
Echolucency	US	Hypoechoic area within the plaque on B-mode (reference = sternocleidomastoid muscle)	Not applicable	In a study of 44 patients with ESUS, an ipsilateral echolucent non-stenotic carotid plaque was found in 50.0% (38)
Impaired cerebrovascular reserve	TCD	<10% increase of blood flow in the ipsilateral MCA while breathing 5% CO_2_ for 2 min.	BOLD-MRI	Not applicable for non-stenotic plaques
Intraplaque hemorrhage	MRI	Intraplaque hyperintensity on T1W FAT SAT (black blood) and 3D-TOF	MRI	In five studies including 162 patients, intraplaque hemorrhage was found in the ipsilateral carotid in 24.4% of cases (13).
Ipsilateral silent brain infarcts	MRI	Non-lacunar hyperintensity of the brain parenchyma, in the territory of the internal carotid artery, visible on T2W and FLAIR, or DWI (if acute)	CT (would appear as a hypodensity)	No data available for patients with ESUS
Lipid-rich necrotic core	MRI	Collection of foam cells, cholesterol crystals and apoptotic cells that appears iso/hyper-intense on T1W and iso/hypo-intense on T2W.	CT, US (although it is difficult to make the difference with intraplaque hemorrhage on these modalities)	No data available for patients with ESUS
Microembolic signals	TCD	Random audible transient increase (variable threshold) of the Doppler signal within the monitored arterial blood flow, generating a high-intensity signal on the doppler imaging (PWV and M-Mode), visible and moving in the direction of the flow. Duration of recording ≥1 h.^c^	Not applicable	No data available for patients with ESUS
Mural thrombus	MRI	Filling defect on contrast MRI, hyperintense signal adjacent to the lumen on T1W	CT, US	In three studies enrolling 94 patients with ESUS, plaque thrombus was identified in the ipsilateral carotid in 6.9% of cases (13).
Neovascularization	CEUS	Enhancement of the plaque on pulse inversion harmonic imaging (microbubbles carried into the plaque by the blood entering the neovessels)	DCE-MRI	No data available for patients with ESUS
Plaque irregularity	MRI	0.3–0.9 mm fluctuations of the surface of the plaque	CT, CEUS	No data available for patients with ESUS
Thin/ruptured fibrous cap	MRI	Disrupted or invisible dark band adjacent to the lumen on 3D-TOF	CEUS	In two studies enrolling 50 patients with ESUS, a thin or ruptured fibrous cap was found in the ipsilateral carotid in 23.6% of cases (13).
Ulceration	MRI	Depression > 1 mm on the surface of the plaque	CTA, CEUS (the threshold is 2 mm in ultrasound studies)	No data available for patients with ESUS

a *The following high-risk features are used less often: juxta-luminal black hypoechoic area and plaque volume assessed by ultrasound, plaque inflammation measured by standardized (18) F-FDG uptake on positron emission tomography-computed tomography, carotid temperature assessed by microwave radiometry*.

b *For simplicity, the description of each high-risk feature is based on its appearance on the imaging modality of choice*.

c *The sound threshold and the number of MES for a positive examination is variable across studies*.

*AHA, American Heart Association; BOLD, blood oxygen level-dependent; CEUS, contrast-enhanced ultrasound; CI, confidence interval; CT, computed tomography; DCE, dynamic contrast-enhanced; ESUS, embolic stroke of undetermined source; FLAIR, fluid-attenuated inversion recovery; MCA, middle cerebral artery; MRI, Magnetic Resonance Imaging; T1W, T1-weighted imaging; T2W, T2-weighted imaging; TCD, transcranial Doppler ultrasound; and 3D-TOF, 3-dimensional time of flight*.

## Challenges of Establishing Causal Link With Stroke

### Puzzling Clinical Associations

Although studies of high-risk features have provided evidence of an association between non-stenotic carotid plaques and brain infarction in patients with ESUS, establishing causality remains challenging in most cases. The dilemma rests on four clinical observations. First, high-risk features are often found in plaques in the absence of related clinical symptoms (19, 40). In a meta-analysis of eight studies enrolling 323 patients with ESUS, a non-stenotic carotid plaque with high-risk features was identified in the contralateral carotid artery in 4.6% of cases (95% CI: 0.1–13.1) (13). Likewise, in a meta-analysis of 64 studies enrolling 20,571 patients with asymptomatic carotid stenosis of various grades, 26.5% of patients were found to have at least one high-risk plaque feature (95% CI: 22.9–30.3). The highest prevalence was observed for neovascularization (43.4%, 95% CI: 31.4–55.8) and the lowest for mural thrombus (7.3%, 95% CI: 2.5–19.4). On average, intraplaque hemorrhage was found in 1 out of 5 patients (19). Second, high-risk plaque features are not specific for symptomatic carotid plaques. In a meta-analysis of data from 20 prospective studies enrolling 1,652 patients with symptomatic carotid stenosis, high-risk plaque features were identified in <1 in 2 patients (43.3%, 95% CI: 33.6–53.2) (19). Third, in patients with stroke, there is an association between the presence of high-risk plaque features and atrial fibrillation. In a study of 68 patients with embolic stroke, including 45 ESUS, the presence of high-risk plaque features on carotid ultrasound (ulceration, thickness ≥ 3 mm, and echolucency) was independently associated with detection of atrial fibrillation on admission or during follow-up (OR = 4.5, 95% CI: 1.0–19.6) (41). Fourth, in some patients with ESUS diagnosed using the current clinical definition, non-stenotic carotid plaques often coexist with other potential causes of stroke, including atrial fibrillation (8.5%) (15), intracranial atherosclerosis (8.4%) (42), PFO (5–9%) (43, 44), and atrial cardiopathy (2.4%) (45).

### Lack of Reliable Biomarkers

The identification of an ipsilateral non-stenotic carotid plaque with or without high-risk features is not sufficient to reclassify ESUS as stroke due to large vessel disease. Further research is, therefore, needed to determine whether combination of vascular imaging findings, clinical data, and candidate biomarkers of plaque progression/instability or atheroembolism (46–82) into multiparameter scores could improve the ability to (1) establish a causal link between ESUS and a non-stenotic carotid plaque, (2) predict plaque progression or stroke recurrence, and (3) select patients who might benefit from adjuvant anti-inflammatory and lipid-lowering therapies as briefly discussed in the next section. Some biomarkers of plaque progression and instability that warrant further investigation specifically in patients with ESUS are presented in Table 3. There are several ongoing projects exploring biomarkers in patients with ESUS or cryptogenic stroke, notably the Searching for Explanations for Cryptogenic Stroke in the Young: Revealing the Etiology, Triggers, and Outcome study (SECRETO, NCT01934725) (95), the Carotid Plaque Imaging in Acute Stroke study (CAPIAS, NCT01284933) (35), and the Biomarkers of Acute Stroke Etiology study (BASE, NCT02014896) (96). Efforts to establish a causal relationship between non-stenotic carotid stenosis and ESUS using biomarkers and multimodal vascular imaging in well-phenotyped prospective cohorts will also benefit from research aiming to identify alternative causes of stroke in patients with ESUS (14, 68, 97–104).

**Table 3:** Biomarkers of potential interest for the study of non-stenotic carotid plaques in ESUS.

**Biomarker**	**Type**	**Main source**	**Key evidence**	**Specific target of a drug previously tested in human trials**	**References**
Lectin-like oxidized LDL receptor 1 (LOX-1)	Protein	Endothelial cells, smooth muscle cells, fibroblasts	In 4,703 participants from the Malmo Diet and Cancer Cohort, higher plasma levels of soluble LOX-1 were associated with higher risk of stroke during a mean follow-up of 16.5 years (HR = 1.5, 95% CI: 1.3–2.4). In 202 patients undergoing carotid endarterectomy, plasma levels of soluble LOX-1 were correlated with the plaque content of oxidized LDL, proinflammatory cytokines, and matrix metalloproteinases.	No	(46–49, 59, 75)
Omentin-1	Protein	Visceral adipose tissue, stromal vascular cells, lung, heart, placenta, ovaries	In 173 patients with acute ischemic stroke, serum levels of omentin-1 were lower in subjects with unstable plaque (*n*= 38, echolucent, thin fibrous cap, ulcerated) than in those with stable plaques (median of 53 vs. 62 ng/mL).	No	(69)
Lipoprotein-associated phospholipase A2 (Lp-PLA2)	Protein	Monocytes, macrophages, T lymphocytes, and mast cells	In 1,946 participants of the Northern Manhattan study, there was a dose-response relationship between Lp-PLA2 mass and the risk of first-ever stroke due to large vessel atherosclerosis (HR = 1.4, 4.5, and 5.1 for quartiles 2, 3, and 4 compared with quartile 1 in multivariable survival analysis).	Yes (Darapladib)	(52, 53, 83)
Chitinase-3-like-1 (YKL-40)	Protein	Inflammatory cells	In 1,132 patients with carotid atherosclerotic plaques of various grades, higher levels of YKL-40 were associated with plaque instability (*n* = 855, echolucency) after adjusting for various demographic and cardiovascular risk factors (OR = 2.1 and 1.7 for quartiles 3 and 4, respectively).	No	(56, 59)
Granzyme B	Protein	T lymphocytes	In 67 patients with severe carotid stenosis undergoing revascularization, higher plasma levels of granzyme B were found in patients with unstable plaques (*n* = 16, echolucent) than in those with stable plaques (median of 492.0 vs. 143.8 pg/mL)	No	(57)
Vimentin	Protein	Endothelial cells, macrophages, and astrocytes	In 4,514 patients with carotid plaques in the Malmo Diet and Cancer Cohort, higher plasma levels of vimentin at baseline were associated with the incidence of ischemic stroke after a mean follow-up of 22 years (HR = 1.66, 95% CI: 1.23–2.25).	Yes (Withaferin-A)	(65, 84)
Macrophage chemoattractant protein (MCP-1/CCL2)	Protein	Monocytes	In the Athero-EXPRESS biobank, higher plaque levels of MCP-1 levels were found in symptomatic (vs. asymptomatic) plaques and in vulnerable (vs. stable) plaques.	No	(61)
Matrix metalloproteinase 9 (MMP9)	Protein	Macrophages, foam cells	Serum levels of MMP9 were higher in large artery atherosclerosis strokes (*n* = 26, 1,137 ng/mL) vs. cardioembolic strokes (*n* = 86, 517 ng/mL). MMP9 >1,110 ng/mL had 85% sensitivity and 52% specificity for differentiating large vessel from cardioembolic strokes.	No	(59, 66)
Complement 5b-9	Protein	Liver	In 70 patients with acute ischemic stroke, serum C5b-9 levels were higher in patients with unstable plaques (*n* = 37) than in those with stable plaques (median of 875 vs. 786 ng/mL). There was also a positive correlation with plaque burden and grade of stenosis.	Yes (Eculizumab)	(76, 85)
Interleukin 1β (IL-1β)	Protein	Monocytes, macrophages	A higher expression of IL-1β and other components of the NLRP3 inflammasome was observed in 30 plaques when compared with 10 healthy mesenteric arteries, both at the protein and the mRNA level.	Yes (Anakinra, Rilonacept, Canakinumab)	(77, 86–88)
Interleukin 6 (IL-6)	Protein	Monocytes, macrophages	In a sub-analysis of data from 703 participants of the population-based Tromsø study, higher plasma levels of IL-6 were independently associated with plaque progression after a 6-year follow-up (OR 1.4, 95% CI 1.1–1.8 per 1 SD increase in IL-6 level).	Yes (Ziltivekimab, Tocilizumab)	(71–74)
C-Reactive Protein (CRP)	Protein	Hepatocytes, white blood cells, adipocytes, smooth muscle cells	In a prospective observational study enrolling 271 participants, higher levels of CRP (quartile 4 vs. 1) were associated with plaque progression after a follow-up of 37 months (OR = 1.8, 95% CI: 1.03–2.99).	No	(78, 89)
CD36	Protein	Various cells including monocytes, endothelial cells, adipocytes, platelets.	In 62 patients with severe carotid stenosis undergoing revascularization, plasma levels of soluble CD36 were higher in those with symptomatic (*n* = 31) and unstable (echolucent, *n* = 20) plaques.	No	(60)
Lipoprotein (a)	Lipoprotein	Food/Liver	In 876 consecutive patients with carotid atherosclerosis (2.5% occlusions), plasma lipoprotein (a) was an independent predictor of carotid occlusion (OR=1.7, 95% CI: 1.2–2.3 per 1 SD increase), suggesting that it plays a role in plaque destabilization/rupture, thrombosis, and impaired fibrinolysis. In 225 patients with coronary artery disease who underwent intra-coronary optical coherence tomography imaging of culprit plaque, the prevalence of thin fibrous cap atheroma was significantly higher in the group with higher serum lipoprotein (a) levels (>25 mg/dL, *n*=87): 23 vs. 11%.	Yes (AKCEA-Apo(a)-LRx)	(79–81, 90, 91)
Non-HDL cholesterol	Lipoproteins	Food/Liver	In 2,888 patients with carotid plaque, including 1,505 with vulnerable plaques (echolucent, irregular, or ulcerated), higher serum levels of non-HDL cholesterol were independently associated with plaque vulnerability (OR = 1.5 for tertile 3 vs. 1, 95% CI: 1.2–1.8).	Yes (various class of lipid lowering drugs)	(51, 92, 93)
Uric acid	Xanthine (purine derivatives)	Various cells	In a study including 88 patients with carotid plaques (44 symptomatic), serum uric acid levels were significantly higher in patients with symptomatic plaques (7.4 vs. 5.4 mg/dL) who also had higher plaque expression of xanthine oxidase as assessed by immunohistochemistry.	Yes (allopurinol)	(82)
Neutrophil count	Cells	NA	In 60 patients with recently symptomatic carotid artery disease, higher neutrophil count (>5,900/μL) was associated with detection of microembolic signals on transcranial Doppler monitoring.	No	(58)
miR-199b-3p, miR-27b-3p, miR-130a-3p, miR- 221-3p, and miR-24-3p	RNA	Various cells	In 60 patients with moderate or severe asymptomatic carotid stenosis, higher plasma levels of the micro-RNAs were associated with plaque progression (*n* = 19) after 2 years of follow-up.	No	(62)
miR-200c	RNA	Various cells	In 22 patients undergoing carotid endarterectomy, higher levels of miR-200c were found in patients with unstable plaques (echolucent symptomatic) and were positively correlated with biomarkers of plaque instability (matrix metalloproteinase—MMP1, MMP9; interleukin 6, macrophage chemoattractant protein 1—MCP-1)	No	(59, 94)
Resistin and chimerin mRNA	RNA	Various cells	In an analysis of 165 carotid plaque (67% unstable based on histological criteria), Resistin and chemerin mRNA expression was 80 and 32% lower, respectively, in unstable vs. stable plaques.	No	(70)

## Challenges of Secondary Stroke Prevention

As a result of the challenges to determine the root cause of an ESUS, the optimal treatment strategy for patients with ESUS remains unclear, and a tailored approach would likely be the most appropriate (9). In this section, we briefly describe the strategies that have been explored so far and discuss possible future directions.

### Dual Antiplatelet Therapy and Antiplatelet Switch

Following the results of the Platelet-Oriented Inhibition in New TIA and Minor Ischemic Stroke (POINT) (105) and the Clopidogrel in High-Risk Patients with Acute Non-disabling Cerebrovascular Events (CHANCE) (106) trials, patients with ESUS are treated with Aspirin-based dual antiplatelet therapy for 21 days provided that their baseline NIHSS is low. After 3 weeks, patients ideally return to single antiplatelet therapy and switching from Aspirin to Clopidogrel is considered in patients who had an ESUS while on Aspirin (107). A meta-analysis of data from CHANCE and POINT showed that extending the treatment beyond 3 weeks might increase the bleeding risk without additional benefit for secondary stroke prevention (108). Whether the presence of ipsilateral non-stenotic carotid plaque with or without high-risk features would modify the magnitude (absolute risk reduction) and duration (beyond 21 days) of the benefits derived from dual antiplatelet therapy in patients with ESUS remains unknown. In patients allergic to Clopidogrel and in carriers of a CYP2C19 loss of function allele, Ticagrelor might be an alternative according to findings of the Acute Stroke or Transient Ischemic Attack Treated with Ticagrelor and ASA [acetylsalicylic acid] for Prevention of Stroke and Death (THALES) trial (109–112). The ongoing Clopidogrel with Aspirin in High-risk patients with Acute Non-disabling Cerebrovascular Events II (CHANCE-2, NCT04078737) trial is evaluating the superiority of the Ticagrelor-Aspirin combination over Clopidogrel-Aspirin therapy in CYP2C19 loss of function carriers with minor stroke or transient ischemic attack (TIA) (113). There is currently no evidence supporting the use of dual antiplatelet therapies not containing Aspirin or triple antiplatelet therapies (with or without Aspirin) for secondary stroke prevention in patients with acute stroke or TIA (114).

### Anticoagulation

The New Approach Rivaroxaban Inhibition of Factor Xa in a Global Trial vs. ASA [Acetylsalicylic Acid] to Prevent Embolism in Embolic Stroke of Undetermined Source (NAVIGATE-ESUS) and the Randomized Double-Blind Evaluation in Secondary Stroke Prevention Comparing The Efficacy Of Oral Thrombin Inhibitor Dabigatran Etexilate for Secondary Stroke Prevention in Patients With Embolic Stroke of Undetermined Source (RE-SPECT-ESUS) trials have shown that universal full-dose oral anticoagulation is not an effective strategy to reduce the risk of stroke recurrence in patients with ESUS (5, 6). These results are likely explained by the heterogeneity of stroke mechanisms in patients with ESUS as discussed earlier, with atrial fibrillation being diagnosed in only 24.8% of cases at 24 months using insertable cardiac monitors (115). Moreover, there is no evidence that patients with ESUS and ipsilateral non-stenotic carotid plaques should be treated differently than those without plaques. In a subgroup analysis of data from 2,905 patients with non-stenotic carotid plaques enrolled in the NAVIGATE-ESUS trial, there was no difference between Rivaroxaban and Aspirin with respect to the prevention of ipsilateral ischemic stroke [Hazard ratio [HR] = 0.6, 95% CI: 0.2–1.9]. Major bleeding complications were significantly more frequent in patients taking anticoagulation (HR = 3.7, 95% CI: 1.6–8.7) (16).

In the Cardiovascular Outcomes for People Using Anticoagulation Strategies (COMPASS) trial, the combination Rivaroxaban-Aspirin (2.5 mg twice daily plus Aspirin 100 mg once per day) was superior to Aspirin alone (100 mg once daily) for the prevention of cardioembolic strokes (HR = 0.4, 95% CI: 0.2–0.8) and ESUS (HR = 0.3, 95% CI: 0.1–0.7) but there was no effect on the incidence of stroke due to moderate-to-severe carotid stenosis (HR = 0.9, 95% CI: 0.5–1.6) (116). Although these results suggest that the combination of Aspirin and low-dose Rivaroxaban could be an effective secondary stroke prevention strategy, they are not directly applicable to patients with ESUS since all patients with acute stroke (<1 month) were excluded from the trial due to the perceived higher risk of major intracranial bleeding (117). Furthermore, the baseline proportion of patients with non-stenotic carotid plaque, with or without high-risk features, was not reported. The prevalence of ipsilateral non-stenotic carotid plaque in participants diagnosed with ESUS during follow-up was also not reported.

According to currently available data, patients with ESUS and features of atrial cardiopathy, notably atrial enlargement, constitute the only subgroup that may benefit from anticoagulation (118). However, since these results are derived from a *post-hoc* analysis of the NAVIGATE-ESUS trial, they might not be used to justify universal prescription of anticoagulation until confirmation is obtained in dedicated trials. The ongoing Atrial Cardiopathy and Antithrombotic Drugs in Prevention After Cryptogenic Stroke (ARCADIA, NCT03192215) (101), Apixaban for Treatment of Embolic Stroke of Undetermined Source (ATTICUS, NCT02427126), and A Study on BMS-986177 (oral factor XIa inhibitor) for the Prevention of a Stroke in Patients Receiving Aspirin and Clopidogrel (AXIOMATIC-SSP, NCT03766581) trials will, hopefully, provide conclusive results to guide patient care. Likewise, in the Oxford Vascular Study, a large patent foramen ovale is present in 36% of patients with a cryptogenic stroke aged >60 years (119) and associated with a 2.5 times higher risk of recurrent ischemic stroke (120), thus suggesting it might be worth trialing PFO closure or anticoagulation in elderly patients with a large PFO. However, the causal relationship between the PFO and the recurrent stroke was not formally established and the prevalence of ipsilateral non-stenotic carotid plaque not reported. Because PFO closure or anticoagulation are not expected to prevent strokes due to large vessel atherosclerosis, trials of PFO closure or anticoagulation in elderly patients with a large PFO should carefully plan subgroup analyses according to the presence of alternative candidate causes of the recurrent stroke, notably an atrial cardiopathy or an ipsilateral non-stenotic carotid plaque that may coexist with PFO (43, 44, 121).

### Other Therapies and Interventions

Currently, patients with ESUS receive intensive lipid-lowering therapy (e.g., statins, ezetimibe) to achieve a level of LDL cholesterol <70 mg/dL (1.8 mmol/L) as early as possible after stroke (122–124). The treatment is maintained long-term if well-tolerated, even in older adults (125–128). Specific targets of LDL cholesterol have not been assessed in patients with ESUS and it is unknown if the presence of an ipsilateral non-stenotic carotid plaque would modify the effect of lipid-lowering drugs as suggested by findings of the Stroke Prevention by Aggressive Reduction in Cholesterol Levels (SPARCL) (129). Furthermore, the potential role of newer classes of lipid-lowering drugs for plaque stabilization and secondary stroke prevention is yet to be defined. Such drugs include proprotein convertase subtilisin/kexin type 9 (PCSK9) inhibitors (small interfering RNA—inclisiran or monoclonal antibodies—evolocumab or alirocumab) and Apo(a) antisense oligonucleotides that reduce plasma levels of both LDL cholesterol and lipoprotein(a) [Lp(a)]; as well as anti- angiopoietin-like 3 monoclonal antibodies that do not affect Lp(a) levels and bempedoic acid (92, 130–135). Like ezetimibe (93, 136), the new lipid-lowering drugs may be useful as add-on or statin-sparing agents in cases of allergy or intolerance to statins, familial hypercholesterolemia, refractory hypercholesterolemia, or in patients with high Lp(a) levels at the time of stroke since statins increase plasma levels of Lp(a) (90, 137). There are reports of an association between high Lp(a) levels and cryptogenic stroke (138, 139) suggesting that Lp(a) could represent a biomarker to guide optimization of lipid-lowering therapy in patients with ESUS as is the case in other cardiovascular diseases.

Systemic inflammation, a hallmark of atherosclerosis, modulates the risk of stroke and the effect of lipid-lowering agents (140–142). This explains the benefit of various anti-inflammatory drugs (e.g., canakinumab, colchicine) for the prevention of atherosclerotic cardiovascular diseases (86, 87, 143). In patients with ESUS and ipsilateral non-stenotic carotid plaque, the effect of anti-inflammatory agents is worth exploring, especially in those with high-risk plaque features since they would not be offered revascularization procedures as first-line treatment according to current guidelines (144–146). Data from the ongoing Colchicine for Prevention of Vascular Inflammation in Non-Cardioembolic Stroke (CONVINCE, NCT02898610) might answer the question of whether patients with ESUS with or without ipsilateral non-stenotic carotid plaques would benefit from the addition of low-dose colchicine to best medical therapy for secondary stroke prevention (147). The relevance of serial vascular imaging to monitor carotid plaque progression and stability is another aspect of the management that remains unexplored.

Besides pharmacological treatments, there is a variety of lifestyle interventions that are beneficial for cardiovascular risk reduction and are recommended by the American Heart Association for secondary stroke prevention no matter the suspected underlying etiology. Such interventions include smoking cessation, regular physical activity, weight loss, improved sleep hygiene, avoidance of noise and air pollution, reduction of salt and sugar intake, higher consumption of fish, fruits, and vegetables (148–155).

## Conclusion

ESUS is a common subtype of stroke that is frequently associated with an ipsilateral non-stenotic carotid plaque. Evidence suggests that advanced multimodal vascular imaging and biomarkers might help reclassify some ESUS as large vessel strokes. However, the precise algorithm for this reclassification remains to be designed. Despite significant research efforts since the term ESUS was coined in 2014, the optimal management strategy for patients with ESUS remains unclear. There are several ongoing trials investigating various interventions. While waiting for more evidence to support the design of tailored therapeutic guidelines for the various well-phenotyped subgroups of patients with ESUS, clinicians should continue to fully implement all previously validated stroke prevention strategies, whether an ipsilateral non-stenotic carotid plaque is present or not. Such strategies include short-term dual antiplatelet therapy if appropriate, long-term intensive lipid lowering therapy, control of modifiable cardiovascular risk factors (e.g., hypertension, diabetes, smoking, obesity), and lifestyle changes.

## Author Contributions

JK-T did the literature search and wrote the manuscript. MV and JK-T prepared the figure. AN, SF, DM, GS, TJ, ES, MV, and GJ critically revised the manuscript. All authors approved the final version.

## Funding

GJ received research grant support from Canadian Institutes of Health Research (CIHR), Heart and Stroke Foundation, University Hospital Foundation, Canada Foundation for Innovation (CFI), and National Institutes of Health (NIH). JK-T was supported by the Faculty of Medicine and Dentistry Motyl Graduate Studentship in Cardiac Sciences, an Alberta Innovates Graduate Student Scholarship, the Ballermann Translational Research Fellowship, the Izaak Walton Killam Memorial Scholarship, and the Andrew Stewart Memorial Graduate Prize.

## Conflict of Interest

The authors declare that the research was conducted in the absence of any commercial or financial relationships that could be construed as a potential conflict of interest.

## Publisher's Note

All claims expressed in this article are solely those of the authors and do not necessarily represent those of their affiliated organizations, or those of the publisher, the editors and the reviewers. Any product that may be evaluated in this article, or claim that may be made by its manufacturer, is not guaranteed or endorsed by the publisher.
